# Attenuation of Cerebral Ischemic Injury in Smad1 Deficient Mice

**DOI:** 10.1371/journal.pone.0136967

**Published:** 2015-08-28

**Authors:** Jamie K. Wong, Lei Chen, Yong Huang, Fatima A. Sehba, Roland H. Friedel, Hongyan Zou

**Affiliations:** 1 Fishberg Department of Neuroscience, Friedman Brain Institute, Icahn School of Medicine at Mount Sinai, New York, NY, 10029, United States of America; 2 Department of Neurosurgery, Icahn School of Medicine at Mount Sinai, New York, NY, 10029, United States of America; School of Pharmacy, Texas Tech University HSC, UNITED STATES

## Abstract

Stroke results in brain tissue damage from ischemia and oxidative stress. Molecular regulators of the protective versus deleterious cellular responses after cerebral ischemia remain to be identified. Here, we show that deletion of Smad1, a conserved transcription factor that mediates canonical bone morphogenetic protein (BMP) signaling, results in neuroprotection in an ischemia-reperfusion (I/R) stroke model. Uninjured mice with conditional deletion of *Smad1* in the CNS (*Smad1* cKO) displayed upregulation of the reactive astrocyte marker GFAP and hypertrophic morphological changes in astrocytes compared to littermate controls. Additionally, cultured *Smad1* ^*-/-*^ astrocytes exhibited an enhanced antioxidant capacity. When subjected to I/R injury by transient middle cerebral artery occlusion (tMCAO), *Smad1* cKO mice showed enhanced neuronal survival and improved neurological recovery at 7 days post-stroke. This neuroprotective phenotype is associated with attenuated reactive astrocytosis and neuroinflammation, along with reductions in oxidative stress, p53 induction, and apoptosis. Our data suggest that Smad1-mediated signaling pathway is involved in stroke pathophysiology and may present a new potential target for stroke therapy.

## Introduction

The restriction of cerebral blood flow during ischemic stroke initiates a stereotypical pathological cascade that culminates in oxidative stress and apoptosis. Reperfusion after ischemia and the neuroinflammation that ensues further exacerbate oxidative damage via production of excessive reactive oxygen or nitrogen species (ROS/RNS) and pro-inflammatory cytokines [1]. Cerebral ischemia also activates astrocytes, the most abundant population of non-neuronal cells in the brain and first responders to ischemic insult [2]. Reactive astrocytes form an astroglial scar that demarcates the infarct area from neighboring tissue, but also constitutes a physical barrier to axon regeneration [3]. Importantly, astrocytes are capable of protecting neurons from ischemic injury by releasing the antioxidant glutathione and taking up extracellular glutamate to combat excitotoxicity [3, 4]. Although the neuronal and glial responses to cerebral ischemia-reperfusion (I/R) injury are well described, the underlying molecular mechanisms remain obscure. Identifying key molecular regulators that can influence stroke outcome remains a pressing issue.

Smad1 is a conserved transcription factor that is activated by canonical bone morphogenetic protein (BMP) signaling [5]. BMPs and BMP receptors are upregulated following CNS injury [6] and implicated in glial scar formation after ischemic stroke [7]. However, the precise functions of BMP/Smad1 signaling in stroke pathophysiology remain unknown; by contrast, its roles in I/R injury outside the CNS have begun to emerge. For instance, following myocardial infarction, BMP4/Smad1 signaling promotes cardiomyocyte apoptosis through ROS-dependent pathways, and inhibition of BMP signaling confers protection against acute myocardial infarction [8, 9]. Moreover, during DNA damage response (DDR), Smad1 is induced and contributes to cellular damage via p53-mediated cell death pathways, and Smad1 inhibition suppresses p53 induction, thereby rendering cells resistant to cell death due to DNA damage [10]. Of note, p53 is a well-characterized apoptotic gene that is upregulated during cerebral ischemia and it promotes apoptosis through regulating cell death-associated target genes (e.g. *Bax*, *Puma*, *Noxa*) or the mitochondria apoptogenic pathway [11, 12]. Additionally, misregulation of BMP signaling is linked to many pathological conditions involving inflammation, oxidative stress and cellular damage [13–15]. These studies raise the possibility that Smad1 may play a pivotal role in mediating cell death and tissue injury following cerebral ischemia. We therefore investigated whether Smad1 deletion would ameliorate I/R injury and improve neurological recovery following transient ischemic stroke.

Here, we report that Smad1-deficient mice exhibit enhanced neuronal survival and improved neurological recovery at 7 days after transient middle cerebral artery occlusion (tMCAO). This is accompanied by attenuated reactive astrocytosis and neuroinflammation, along with reductions in oxidative stress, p53 induction, and apoptosis. Smad1 therefore represents a potential novel target for stroke therapy.

## Materials and Methods

### Generation of Mutant Mice

*Smad1* ^*flox/flox*^ mice [16] and Nestin-Cre mice [17] were obtained from the Jackson laboratory. *Smad1* heterozygous germline deletion (*Smad1* ^+/-^) and *Smad1* cKO (*Smad1* ^*fl/-*^;Nestin-Cre) mice were generated in our laboratory as described previously [18]. Mutant mice and their littermate controls of matched sex underwent tMCAO or sham surgery and were analyzed at various time points for histology, gene expression, and behavioral testing. Both sexes were represented in the control and mutant cohorts. Heterozygote littermates (*Smad1* ^fl/-^ or *Smad1* ^fl/+^;Nestin-Cre) showed similar stroke responses as *Smad1* ^fl/+^ or wildtype mice and were therefore included in the control cohort.

All animal experiments were conducted in accordance with the guidelines and protocols approved by the Institutional Animal Care and Use Committee (IACUC) at Icahn School of Medicine at Mount Sinai.

### Transient MCAO Model

Adult mice (8–12 weeks old) were anesthetized with 110 mg/kg ketamine and 10 mg/kg xylazine. The left common carotid artery (CCA) was exposed and a silicon-coated 7–0 nylon suture was inserted into the internal carotid artery (ICA) to occlude blood flow into the middle cerebral artery (MCA) for 1 hour. Cerebral blood flow (CBF) was monitored using a laser Doppler Flowmetry (LDF) probe (0.8 mm diameter, model P-433, Vasamedics Inc.) that was secured with stereotactic guidance to the temporal area, above the vascular territory of the left MCA and away from large meningeal vessels. Successful MCA occlusion (MCAO) was confirmed by over 80% reduction in the CBF relative to baseline (S6 Fig). After 1 hour, the suture was removed to allow reperfusion, as confirmed by restoration of CBF (S6 Fig). Mice were allowed to survive for 1 week, 1 month, or 3 months post surgery. Overall mortality from the tMCAO surgery was 10%. A separate cohort of older mice (8–10 months old) also underwent tMCAO surgery to investigate whether the effect of *Smad1* deletion on neurological recovery is age-dependent.

For assessment of neurological deficits, the modified Bederson Score scale [19] was applied as follows: loss in consciousness and/or spontaneous activity (score of 4), falling to paretic side (3), circling (2), failing to extend the forepaw of the paretic side fully (1), and no detectable neurologic deficits (0).

For immunohistochemical analyses, we observed similar findings in both sexes; therefore the data from both sexes were combined. For behavioral studies, we also grouped both sexes together for each time point to increase the cohort size for sufficient power for statistical analysis. Specifically, the numbers of littermate mice used were as follows: for IHC analyses at 7 days post-stroke, the control group included 2 females and 2 males, and the *Smad1* cKO group included 1 female and 3 males. For behavioral studies, at 7 days post-tMCAO, control cohort included 4 females and 1 male, and *Smad1* cKO cohort included 4 females and 2 males. For the 3-month behavioral data, the control cohort included 1 female and 3 males, and the *Smad1* cKO cohort included 1 female and 2 male mice.

### Western Blot

Lysates from freshly collected cortical tissue were prepared in RIPA buffer (Sigma), supplemented with protease and phosphatase inhibitors (Sigma), and homogenized using a Dounce homogenizer (Kimble Chase). Protein levels were quantified using BCA assays (Pierce Thermo Scientific). Proteins were resolved on NuPAGE gels (Invitrogen) using the XCell SureLock system (Invitrogen). The immunoreactive bands were detected by fluorescent ODYSSEY infrared imaging system (LI-COR). Equal protein loading was controlled by probing the blots with an antibody to β-actin. Antibodies used were rabbit anti-Smad1 (Abcam, 1:1000) and mouse anti-β-actin (Thermo Scientific, 1:2000).

### Histology and Cell Quantification

Following behavioral analysis, mice were administered an overdose of Euthasol and transcardially perfused with PBS followed by 4% paraformaldehyde (PFA) in PBS. For visualization of vasculature, mice were perfused with 5 ml PBS followed by 2 ml bromophenol blue solution (10 mg/ml in PBS).

Brains were post-fixed overnight, cryoprotected in 30% sucrose, embedded and sectioned in the coronal plane at 20 μm thickness using a cryostat. For immunostaining, sections were collected free-floating in PBS with 0.01% sodium azide. Tissue sections were washed in PBS with 0.1% Triton X-100 (Sigma) and incubated in blocking buffer (10% heat inactivated goat serum, Invitrogen) for 1 hour at room temperature. After overnight incubation with primary antibody at 4°C, sections were washed and then incubated with fluorescent secondary antibodies (Molecular Probes, 1:750) for 2 hours at room temperature. After washing, sections were counterstained with DAPI (Invitrogen, 1:1000), mounted onto Superfrost Plus slides and coverslipped with Fluoromount (EMS).

Primary antibodies used for immunohistochemistry include: rabbit GFAP (Dako Z0334, 1:800), mouse Nestin (Abcam ab6142, 1:100), chicken Vimentin (Novus Biologicals NB300-223, 1:2000), rabbit S100 (Dako Z0311, 1:400), mouse NeuN (Millipore MAB377, 1:200), rabbit Iba1 (Wako 019–19741, 1:500), rabbit activated Caspase 3 (R&D Systems AF835, 1:400), mouse 8-hydroxyl-2’-deoxyguanosine (8-oxo-dG) (Trevigen 4354-MC-050, 1:350), rabbit p53 (Santa Cruz sc6243, 1:50), rat CD31 (BD 553370, 1:250), rabbit Olig2 (Millipore AB9610, 1:500), and chicken GFP (Aves Labs GFP-1020, 1:500). Cresyl violet staining was performed according to the manufacturer’s protocol (EMS).

Images were captured using an Axio Imager.A2 (Zeiss) microscope equipped with an AxioCam MRc. Cell quantification was performed using ImageJ software. Specifically, images of the peri-infarct area in the ipsilateral somatosensory cortex, as well as the corresponding cortical region on the contralateral hemisphere were used for quantification of GFAP^+^ astrocytes, S100^+^ astrocytes, Iba-1^+^ microglia, and NeuN^+^ neurons. Images of the stroke core were also used for quantification of NeuN^+^ neurons. Images sampled throughout the entire ipsilateral hemisphere were used for quantification of activated Caspase 3, 8-oxo-dG, and p53. A minimum of 3 images taken between approximately 1mm rostral and 2mm caudal to Bregma were quantified per animal. All quantifications were performed on photos taken at 10X magnification, except for NeuN and 8-oxo-dG, which were performed on 20X images. Cells in the entire field for each photo were quantified. Quantification was carried out by an experimenter blinded for the genotypes.

### Astrocyte Cultures and MTT Assay

Primary astrocytes were derived from neonatal (P1-P2) mouse cortex. Forebrains were removed aseptically and cells from the neocortex were manually dissociated and seeded at a density of 10^5^/cm^2^ in Dulbecco’s modified Eagle’s medium (DMEM, Gibco) supplemented with 10% FBS (Lonza) and 100U/ml penicillin/streptomycin. Over 95% of cultured cells were identified as astrocytes by positive immunostaining for GFAP and S100. After reaching confluence, astrocytes were trypsinized and seeded in 96 well plates at a density of 10,000 cells/well. Twenty-four hours after passage, cells were challenged with hydrogen peroxide (H_2_O_2_) (50μM) for 6 hours, and cell viability was evaluated using a MTT assay. MTT solution was prepared (5mg/ml in PBS, Sigma) and filtered through a 0.2μm filter. 10 μl of MTT solution was added to 100 μl of DMEM without phenol red to each well. Cells were incubated in a cell culture incubator with 5% CO_2_ at 37°C for 4 hours. The MTT solution was then removed and the formed MTT formazan was dissolved with 200 μl DMSO. Optical density (OD) was determined using a plate reader at 570 nm, with a reference wavelength of 630 nm.

### HEt Assay

Superoxide production in post-stroke brains was detected with *in vivo* hydroethidine labeling (HEt, Invitrogen). HEt was injected intraperitoneally (200μl, 1mg/ml in PBS) 3 hours before perfusion. HEt is oxidized by superoxide to fluorescent ethidium, which intercalates in DNA and fluoresces red.

### Real-time qRT-PCR

RNA was extracted from cortical tissues using RNeasy Mini kit (Qiagen), reverse transcribed (SuperScript III, Invitrogen) and used for qRT-PCR (SYBR Green FastMix, Quanta). Data were normalized to the housekeeping gene *Tfrc* transferrin receptor using the ΔΔCt method. Primers are available upon request. At least three independent experiments with technical triplicates were performed for each condition.

### Statistical Analysis

Neurological scores were analyzed using a repeated measures two-way ANOVA with Bonferroni post-hoc analyses. Astrocyte viability MTT data were analyzed using a one-way ANOVA followed by the Bonferroni test. Student’s t-tests and one-way ANOVA followed by the Bonferroni test were performed for cell count analyses. Data are presented as mean ± SEM, *p<0.05; **p<0.01; and ***p<0.001.

## Results

**Fig 1:**
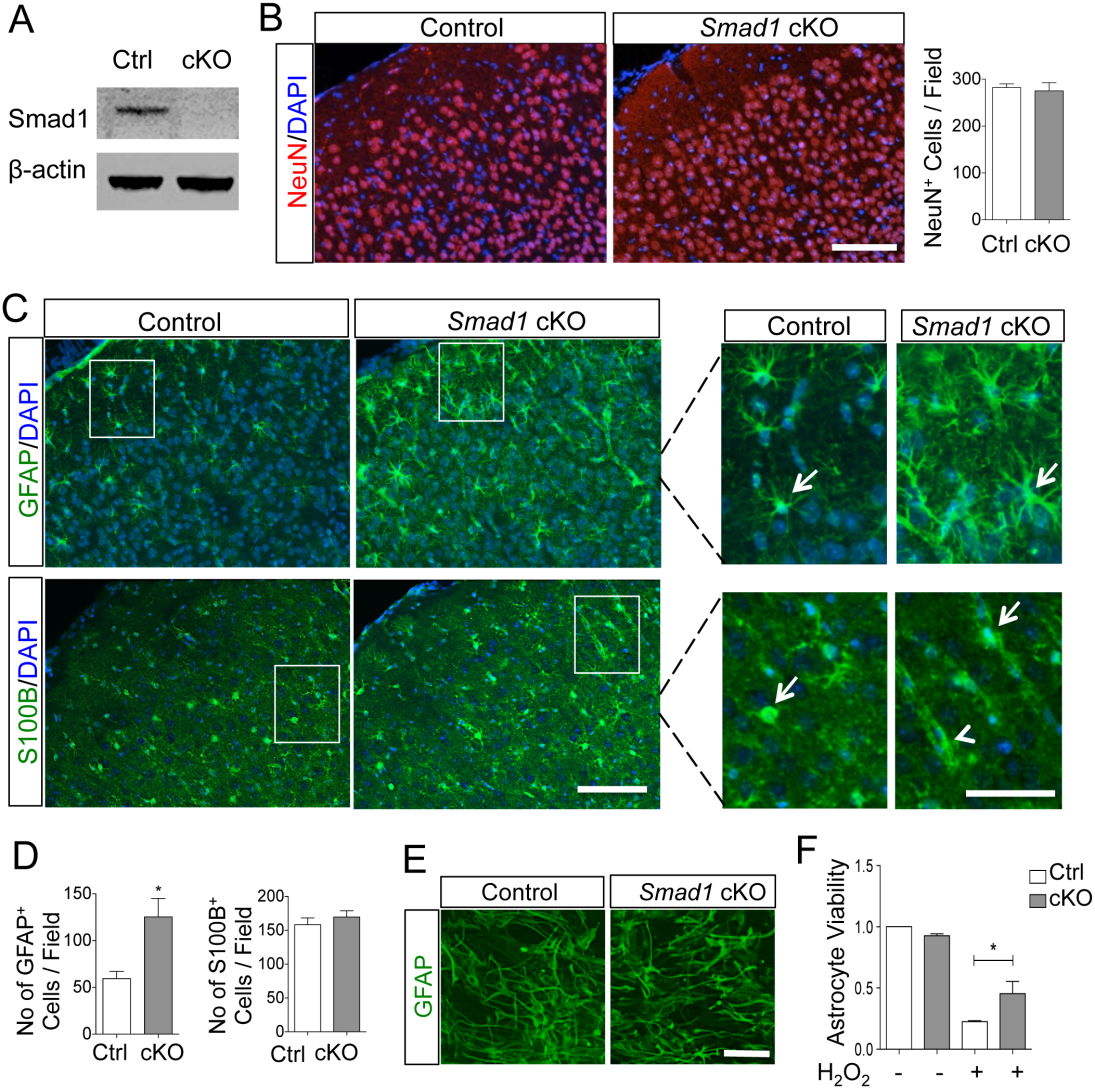
Astrocytes in uninjured *Smad1* cKO mice display increased baseline reactivity and enhanced antioxidant capacity. (A) Western blot analysis confirmed Smad1 knockout in cortical tissue of *Smad1* cKO mice. (B) Images and quantification of IHC revealed no obvious differences in the number or cortical organization of neurons (NeuN^+^) between *Smad1* cKO and littermate controls. (C) Upregulation of GFAP in cortical astrocytes in *Smad1* cKO mice as compared to controls. IHC for S100B, a pan-astrocyte marker for both reactive and non-reactive astrocytes, showed no differences in the number or spatial distribution of astrocytes between mutant and control mice. Of note, the S100 antibody reacts strongly with S100B, and weakly and very weakly with S100A1 and S100A6, respectively, therefore, the immunoreactivity predominantly reflects S100B. Enlarged images of boxed areas in (C) are shown on the right, demonstrating marked hypertrophy of cellular processes of GFAP^+^ astrocytes in *Smad1* cKO mice, indicative of reactive state (arrows). S100B^+^ astrocytes frequently ensheath blood vessels (arrowhead) and also display subtle hypertrophic morphology in the mutant mice (arrows). (D) Quantification of the number of GFAP+ or S100B+ astrocytes per field examined (n = 3, unpaired Student’s t test, *p = 0.01). (E, F) Cultured astrocytes derived from *Smad1* cKO neonatal pups displayed similar expression of GFAP, but exhibited greater tolerance to H_2_O_2_ oxidative stress than control astrocytes by MTT viability assay (n = 3, One-way ANOVA) *p<0.05. Scale, 100 μm (B and C) and 50 μm (E and enlarged photos in C).

To study the role of Smad1 in cerebral ischemia, we generated *Smad1* conditional knockout mice (cKO) with *Smad1* ablation in all neural cells in the CNS (*Smad1* ^*fl/-*^; Nestin-Cre). Western blot analysis confirmed deletion of Smad1 in the CNS in the *Smad1* cKO mice (Fig 1A). The specificity of Nestin-Cre recombination was verified by immunohistochemistry (IHC) in the Rosa26-YFP reporter line, which showed overlap of YFP signal with that of neural markers (S1 Fig), but not of microglial (Iba1^+^) nor endothelial markers (CD31^+^)(S2 Fig). Of note, Cre expression in Nestin-Cre transgenic mice [17] is under the control of the 2^nd^ intron of the rat Nestin gene, which functions as a nervous system-specific enhancer [20]. Hence, the Cre expression pattern in our mutant mice appeared more restricted than that of endogenous Nestin [21]. For instance, endogenous Nestin is also expressed in endothelial cells, however, in our reporter line, Cre-mediated YFP expression was not readily detectable in endothelial cells in the brain (S2B Fig).

### Astrocytes in uninjured *Smad1* cKO mice exhibit increased reactivity

*Smad1* cKO mice developed normally with no significant differences in body weight (S3A Fig) or motosensory behavior compared to littermate controls. The number of cortical neurons and the cortical layout also appeared comparable between genotypes (Fig 1B). Interestingly, IHC revealed an upregulation of GFAP in the cortex of *Smad1* cKO mice in comparison to control littermates (125 ± 20 vs. 59 ± 8 GFAP^+^ cells per field examined, p = 0.01) (Fig 1C and 1D). GFAP is considered to be a sensitive and reliable marker for reactive astrocytes [22], our results therefore suggest increased baseline levels of astrocyte reactivity in the *Smad1* cKO animals. Consistent with this model, the GFAP-expressing astrocytes also displayed marked morphological changes with hypertrophic cellular processes, another well-established measure of astrocyte reactivity (Fig 1C) [22]. To rule out a change in the total number of cortical astrocytes, we performed immunolabeling for S100B, a pan-astrocyte marker for both reactive and non-reactive astrocytes. We found no apparent differences in the number nor spatial distribution of S100B-expressing astrocytes between *Smad1* cKO mice and controls (Fig 1C). Notably, consistent with previous reports [23, 24], S100B appeared more abundantly expressed in a subtype of mature astrocytes ensheathing blood vessels (Fig 1C). There was a subtle hypertrophic change in a subpopulation of S100B-expressing astrocytes in the mutants, which is not as prominent as seen in GFAP-expressing astrocytes (Fig 1C). Of note, S100B, a member of S100 family of EF hand calcium binding proteins, is localized predominantly in central cytoplasm and nucleus, thus does not delineate well cellular processes [23, 24]. In both genotypes, GFAP^+^ astrocytes similarly formed extensive contacts with cortical microvasculature, although mutant GFAP^+^ astrocytes displayed more elaborate cellular processes (S3B Fig). Hence, despite increased GFAP expression, general astrocyte development does not appear to be significantly altered by *Smad1* deletion.

Since astrocytes possess antioxidant capacity, we investigated whether this property might be affected in *Smad1* ^-/-^ astrocytes. Primary astrocytes derived from both mutant and control littermates appeared comparable in cell morphology and GFAP expression (Fig 1E). However, *Smad1* ^*-/-*^ astrocytes displayed significantly enhanced resistance to oxidative stress induced by H_2_O_2_ treatment, as revealed by a 3-(4,5-dimethylthiazol-2-yl)-2,5-diphenyltetrazolium (MTT) assay (viability of 0.45 ± 0.10 vs. 0.23 ± 0.01 in mutant vs. control astrocytes; p < 0.05; Fig 1F). It is noteworthy that CNS tissue is well known for an adaptation process of the so-called ischemic preconditioning effect, whereby a prior small ischemia would lead to protection against subsequent ischemia [25]. Hence, akin to the preconditioning effect, the increased astrocytic reactivity in uninjured *Smad1* cKO mice (reflected by GFAP upregulation and morphological alterations) may lead to enhanced antioxidant capacity in astrocytes as a result of an adaptive process or a priming effect.

### Reduced reactive astrocytosis in *Smad1* cKO mice after transient MCAO

**Fig 2:**
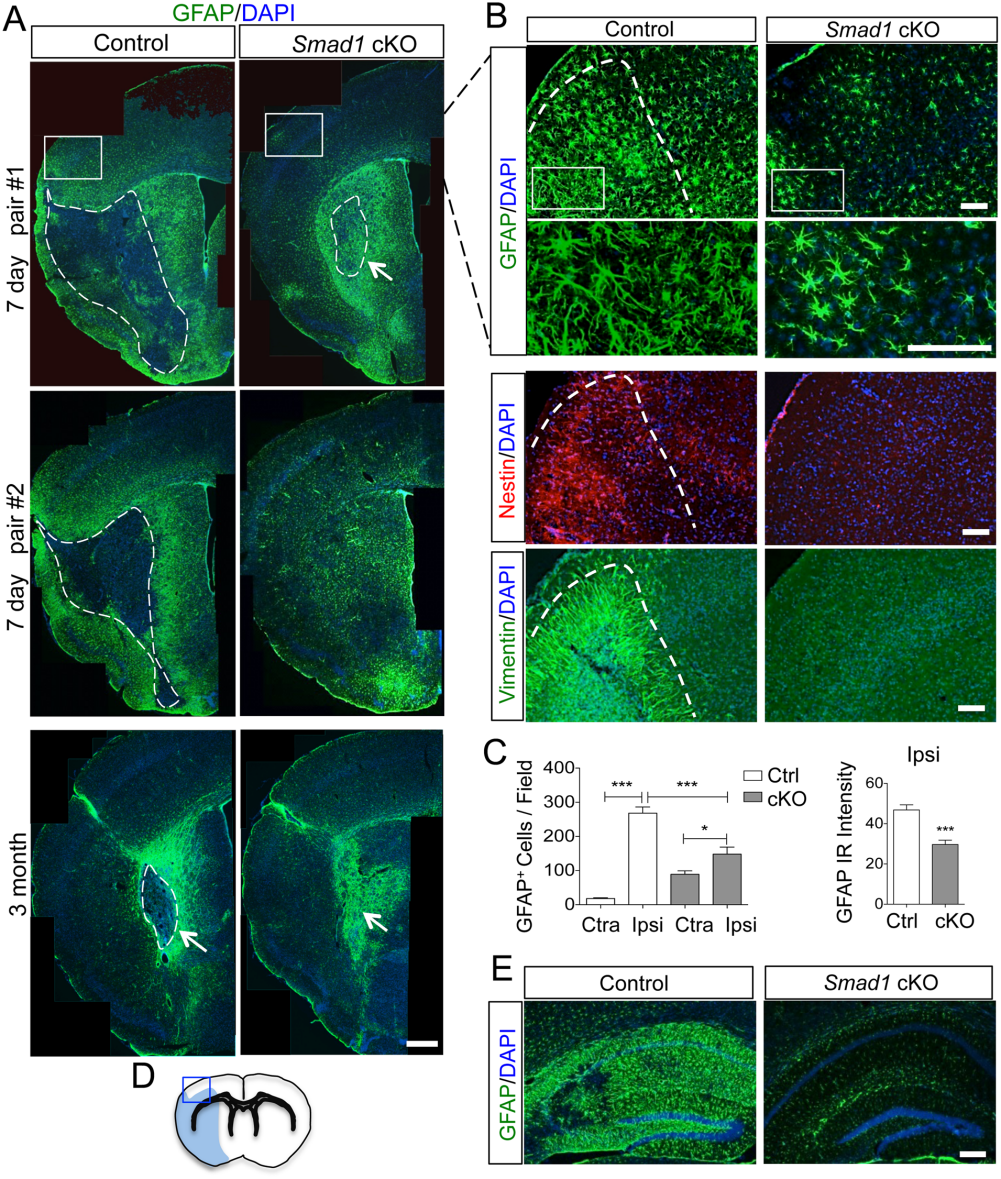
Attenuated reactive astrocytosis after stroke in *Smad1* cKO mice. (A) Images of IHC for reactive astrocyte marker GFAP on ipsilateral hemisphere at 7 days (top two panels) or 3 months (bottom panels) post-stroke. The border between the stroke core and peri-infarct area is outlined by dotted lines. Arrows point to striatal infarct core. (B) Enlarged IHC images of boxed areas in (A) at the cortical peri-infarct area (blue box in D) with the indicated reactive astrocyte markers GFAP, Nestin, and Vimentin. Enlarged images of GFAP IHC highlight the hypertrophic morphology of GFAP^+^ astrocytes in mutants. (C) Quantification of the number of GFAP^+^ astrocytes and the intensity of GFAP immunoreactivity (IR) at the peri-infarct area shown in (B). n = 4, one-way ANOVA for the number of astrocytes, unpaired Student’s t-test for GFAP intensity, ***p<0.001, Ipsi, ipsilateral; Ctra, contralateral cortex. (D) Diagram of infarct territory affected by tMCAO (blue) and peri-infarct cortical area (blue box). (E) Reactive astrocytosis was similarly attenuated in the ipsilateral hippocampus of *Smad1* cKO mice. Scale, 500 μm (A), 100 μm (B), and 200 μm (E).

**Fig 3:**
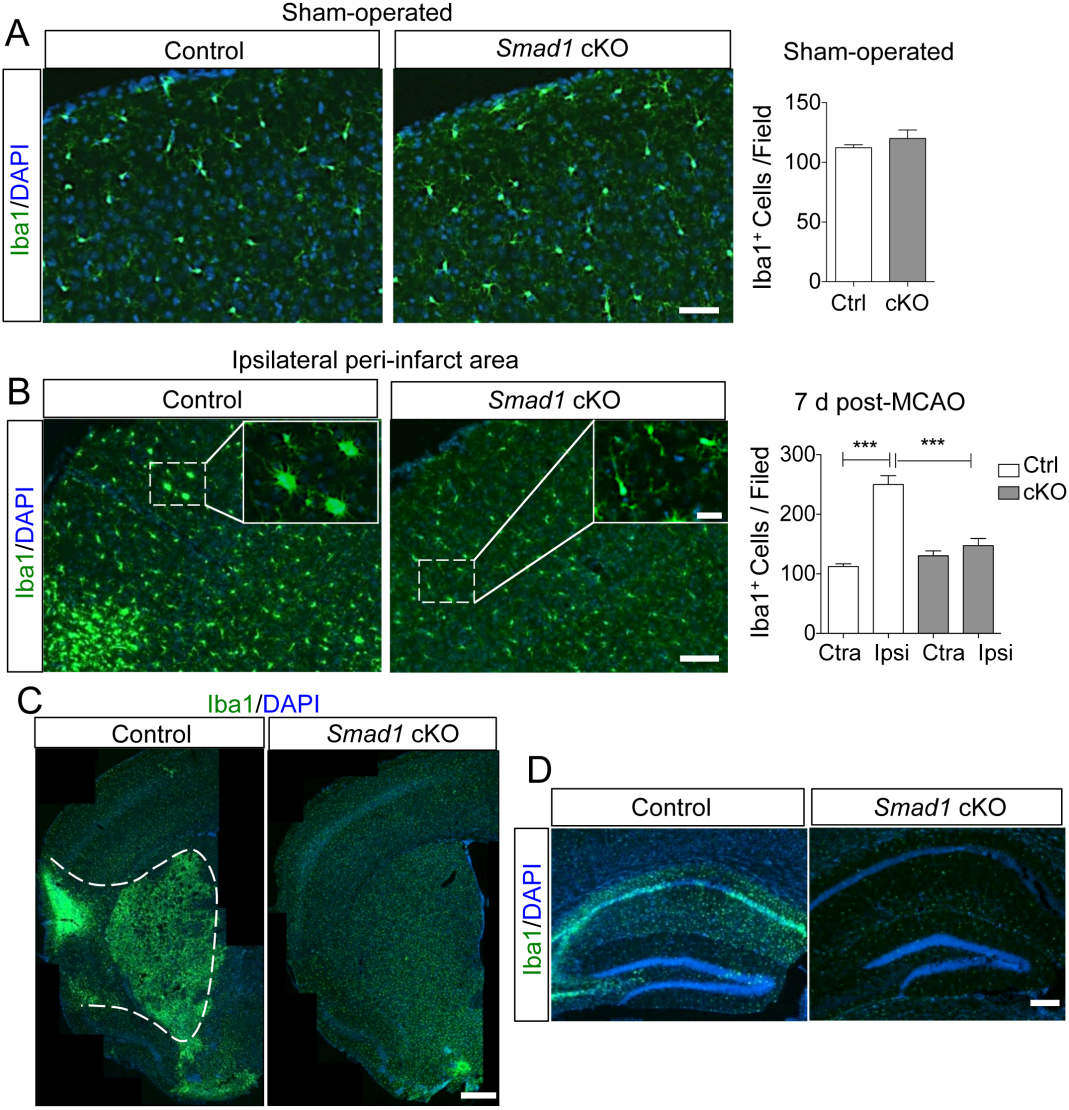
Decreased neuroinflammation following ischemic stroke in *Smad1* cKO mice. (A-B) IHC images and quantification of Iba1^+^ inflammatory cells in the frontal cortex of sham-operated mice (A) or in the cortical peri-infarct area (B) at 7 days post-tMCAO. No difference was detected at baseline (A), but fewer Iba1^+^ cells were found in the cortical peri-infarct area in mutants compared to controls after stroke (B). Differences in morphology of inflammatory cells are shown in enlarged images in insets. n = 3 pairs for sham-operated, unpaired Student’s t-test. n = 4 for 7 days post-MCAO, one-way ANOVA, ***p<0.0001. (C-D) IHC images show reduced inflammation in the ipsilateral striatum (C) and hippocampus (D) in *Smad1* cKO mice at 7 days post-stroke. Ipsi, ipsilateral cortex; Ctra, contralateral cortex. Scale, 100μm (A, B), 25μm (B insets), 500μm (C), and 200μm (D).

Since *Smad1* ^*-/-*^ astrocytes show enhanced antioxidant capacity, and astrocytes can protect against ischemic injury by releasing the antioxidant glutathione [3, 4], we next sought to test whether *Smad1* deletion would attenuate stroke pathophysiology. We performed transient middle cerebral artery occlusion (tMCAO) on adult (8 to 12 weeks old) *Smad1* cKO mice and littermate controls. By day 7 following tMCAO, the infarct core in the control animals could be easily distinguished from the peri-infarct area by a rim of reactive astrocytes (GFAP^+^) and infiltration of inflammatory cells (Iba1^+^) in the frontoparietal cortex, striatum and hippocampus (Figs 2 and 3). In contrast, both astroglial and inflammatory responses were significantly attenuated in *Smad1* cKO mice (Figs 2 and 3). Quantification showed that the number of GFAP^+^ astrocytes in the peri-infarct frontal cortex was decreased by 45% in *Smad1* cKO mice compared to controls (148 ± 21 vs. 268 ± 18 per field examined; p = 0.0003; Fig 2B–2D). The reactive astrocytes in the mutant animals also displayed fewer hypertrophic processes (Fig 2B) and a 50% decrease in the intensity of GFAP immunosignals compared to controls (Fig 2C). IHC for Nestin and Vimentin, two additional markers of reactive astrocytes, confirmed the attenuated astrogliosis in the peri-infarct area of *Smad1* cKO mice (Fig 2B). Similar findings were observed in the ipsilateral hippocampus (Fig 2E). Consistent with an attenuated reactive astrocytosis, the area of tissue damage as outlined by the intense rim of GFAP immunoreactivity (Fig 2A) relative to the area of the ipsilateral cerebral hemisphere was significantly smaller in mutants than controls (6.7% ± 1.9 vs. 30.5% ± 2.6; p < 0.001). It is worth mentioning that although 2,3,5-triphenyltetrazolium chloride (TTC) staining is often used to evaluate infarct size, particularly in permanent MCAO models, it does not reliably distinguish the border between healthy and dead tissue at 7 days after tMCAO. This is because the optimum time to perform TTC staining in the tMCAO model is 24 h to 48 h after ischemia, after which macrophages will infiltrate the infarct area, resulting in smudging of the infarct border and interference with measurement of the infarct volume [26]. In fact, comparative studies have demonstrated that at time points beyond 24 h conventional histopathological analyses are superior to TTC staining for accurate delineation of structural and cellular changes within the infarct tissue [27].

It is notable that *Smad1* ^-/-^ astrocytes did mount a response to ischemic insult, resulting in a 1.7-fold increase in the number of GFAP^+^ astrocytes in the ipsilateral peri-infarct area relative to the contralateral cortex (148 ± 21 vs. 89 ± 10 per field examined; p < 0.05, Fig 2C). However, this astrocytic reaction occurred at a much lower level than in control mice, which showed a 14.9-fold increase in the number of GFAP^+^ astrocytes in ipsilateral than contralateral peri-infarct cortex (268 ± 18 vs. 18 ± 2; Fig 2C). Consistent with a baseline increase in astrocyte reactivity in uninjured mutant mice (Fig 1C), the number of GFAP^+^ astrocytes in the contralateral hemisphere was higher in mutants than controls after stroke (89 ± 10 vs. 18 ± 2, p < 0.001) (Fig 2C). It is also noteworthy that in areas of severe ischemia, such as in the striatal stroke core, which is characteristic of the intraluminal MCA occlusion model used in this study, robust reactive astrocytosis did occur in both mutant and control mice (Fig 2A).

We also examined the astroglial scar at 3 months post-tMCAO. In both control and *Smad1* cKO animals, residual astroglial scarring was visible, but confined to the striatal stroke core, with small necrotic centers sometime observed in control animals (Fig 2A). In both groups, astrocytic reaction had subsided significantly in the cortex by 3 months post-stroke (Fig 2A).

### Microglial activation is attenuated in *Smad1* cKO mice after stroke

At baseline, we observed that Iba1^+^ microglia in the cortex were similar in number and morphology in mutant and control animals (Fig 3A). In response to stroke, in control mice, activated microglia typically display an amoeboid morphology with shortened processes, but this morphological change was less prominent in mutants (Fig 3B). Additionally, at day 7 post-tMCAO, the number of Iba1^+^ inflammatory cells in the peri-infarct cortical area in *Smad1* cKO mice was only 59% of the number present in controls (147 ± 12 vs. 250 ± 15; p < 0.001; Fig 3B). The ipsilateral striatum and hippocampus of mutant mice were also largely spared of major inflammatory changes (Fig 3C and 3D).

Nestin-Cre driven *Smad1* deletion is not expected to occur in microglia, as confirmed by the absence of Cre-mediated recombination in Iba1^+^ cells in the Rosa26-YFP reporter line (S2A Fig). Hence, the reduced inflammatory response in *Smad1* cKO mice is more likely a result of attenuated stroke pathophysiology than microglial dysfunction. We surveyed the gene expression of a number of cytokines and chemokines—*CCL2*, *CCR2*, *CCL5*, *CXCL10*, *CXCR3*, *IFNγ*, *TNFα*, *IL1β and IL6*—in the ischemic cortex at 3 days post-stroke, but did not find statistically significant differences between mutants and controls (S4A Fig), thus future studies on protein levels or spatial distribution will be required to determine potential differences in the cytokine profiles between the two groups.

### *Smad1* deletion is neuroprotective against cerebral ischemia

**Fig 4:**
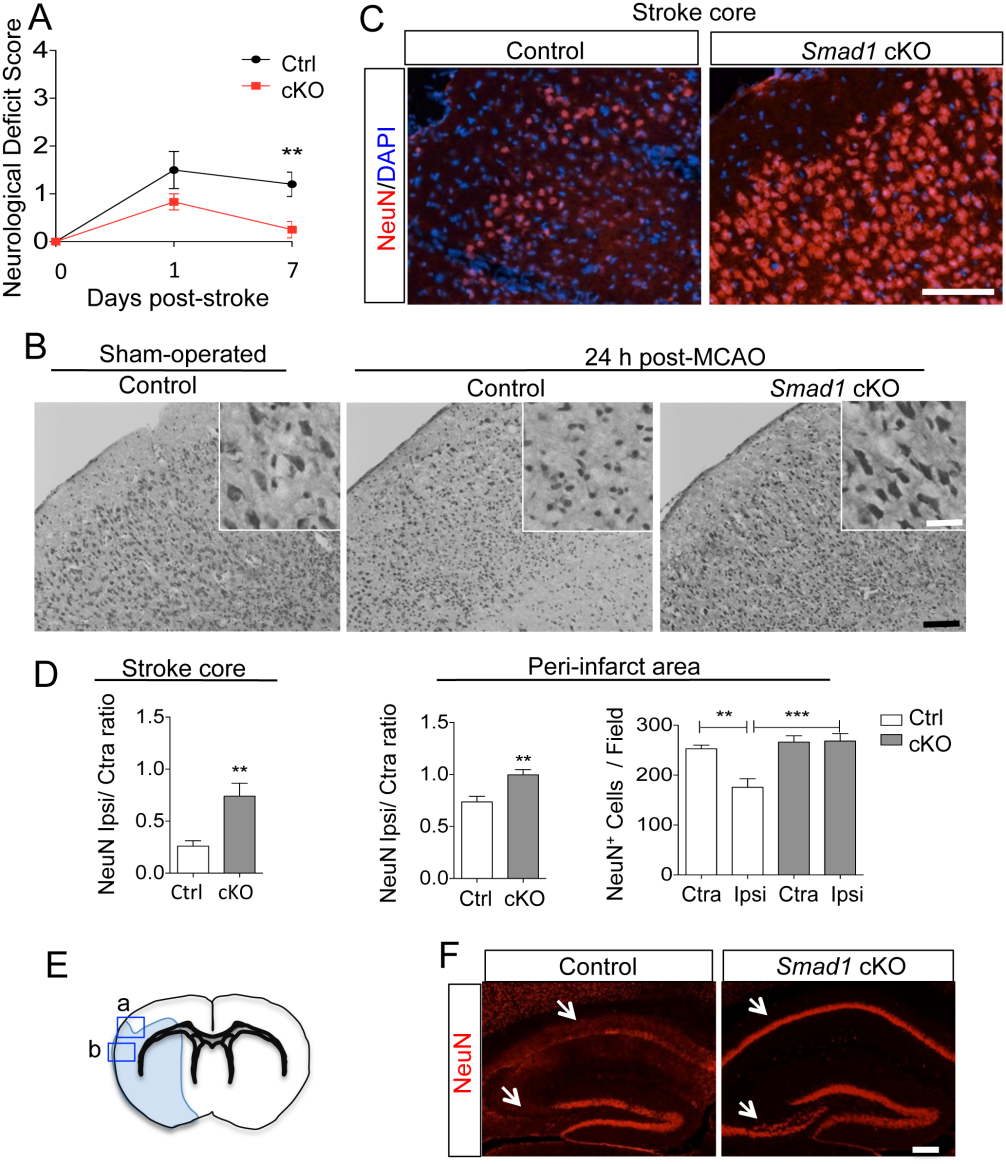
*Smad1* deletion enhances neuroprotection and improves neurological recovery after stroke. (A) *Smad1* cKO mice exhibited significantly improved neurological function at 7 days post-stroke in comparison to controls (n = 4 females and 1 male in the control cohort, and 4 females and 2 males in the *Smad1* cKO cohort. **p<0.01, F = 7.121 for 1 degree of freedom, repeated measures two-way ANOVA). (B) Cresyl violet staining revealed stereotypical attributes of cellular damage in cortical neurons in controls, but less so in *Smad1* cKO mice at 24 h post-stroke. Enlarged images are shown in insets. (C-E) Images of IHC and quantification of the number and ratio of NeuN^+^ neurons in ipsilateral vs. contralateral frontal cortex in the stroke core (box b in E) or cortical peri-infarct area (box a in E) at 7 days post-stroke (n = 4, unpaired Student’s t test for ratios, one-way ANOVA for number of neurons, **p<0.01, ***p<0.001). (F) Less neuronal loss was observed in ipsilateral hippocampus of *Smad1* cKO mice. Arrows point to CA1 and CA3 neurons. Scale, 100 μm (B and C), 25 μm (B insets), and 200 μm (F).

We next investigated whether the attenuated reactive astrocytosis and the reduced neuroinflammation after tMCAO are associated with neuroprotection in mutant mice. Following tMCAO, both groups exhibited comparable neurological deficits at 24 h post-stroke, suggesting similar primary injury in both cohorts (neurological deficit scores of 0.83 ± 0.17 vs. 1.50 ± 0.39; p > 0.05, Fig 4A). However, by 7 days post-stroke, neurological recovery was significantly better in mutant than control animals (neurological deficit scores of 0.25 ± 0.17 vs. 1.20 ± 0.25, Fig 4A). Whereas control mice continued to display asymmetrical posturing, circling, and ptosis of the ipsilateral eyelid, these deficits were either absent or attenuated in *Smad1* cKO mice (S5A Fig). Importantly, at 1 and 3 months post-stroke, there was no sign of delayed neurological deterioration in *Smad1* cKO mice (S5B Fig), in agreement with the immunohistological analyses at 3 months post-tMCAO which revealed that astrogial reaction had largely subsided in the cortex in both cohorts, and only residual astroglial scarring was visible and confined to the striatal stroke core (Fig 2A). Since age can greatly influence stroke outcome [28], we conducted additional tMCAO studies to analyze neurological recovery in older cohorts of mice. We found that *Smad1* cKO mice at 8 to 10 months of age also exhibited significant improvement in functional recovery at 7 days post-stroke (S5C Fig).

We next examined neuronal survival after stroke. Cresyl violet staining at 24 h post-stroke confirmed the stereotypical morphological changes of acute neuronal damage in control animals, including shrunken cell bodies, pyknotic nuclei, perineuronal vacuolations, and neuronal necrosis, all of which were less prominent in *Smad1* cKO mice (Fig 4B). At 7 days post-tMCAO, mutant mice exhibited a 1.5-fold increase in the number of surviving neurons (NeuN^+^) in the peri-infarct cortical area compared to controls (268 ± 15 vs. 176 ± 17, p = 0.001), and a 3-fold increase in the infarct core (194 ± 31 vs. 67 ± 14, p<0.01; Fig 4C–4E). The nuclei of NeuN^+^ neurons appeared pyknotic in control animals, but relatively normal in size and shape in mutants (Fig 4C). In addition, CA1 and CA3 neurons in the ipsilateral hippocampus also exhibited less neuronal damage in mutants (Fig 4F).

### *Smad1* deletion reduces oxidative stress and apoptosis after cerebral ischemia

**Fig 5:**
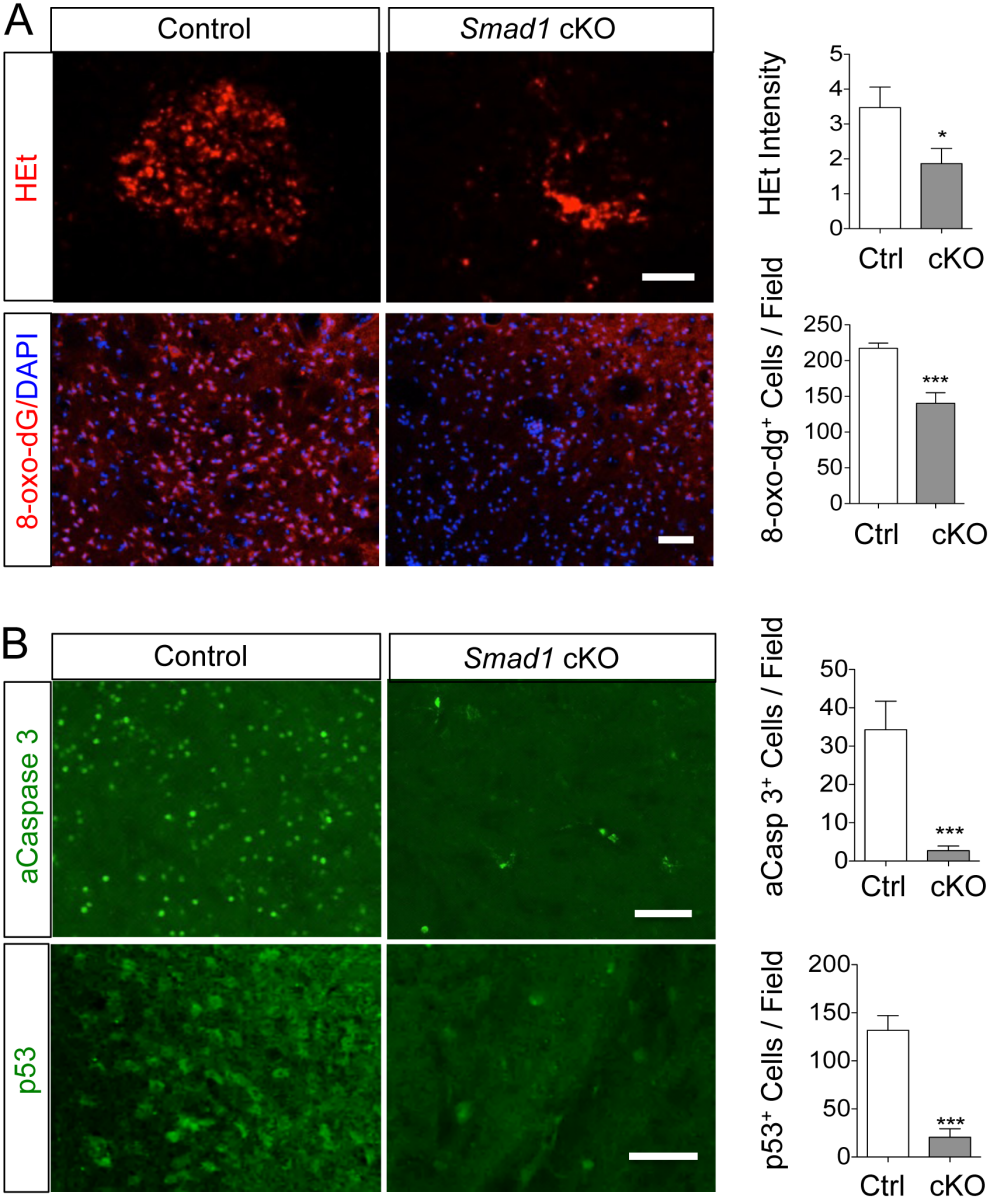
*Smad1* deletion reduces oxidative stress and apoptosis following stroke. (A) Fluorescent images and quantification of *in vivo* HEt assay reflecting ROS levels on the ipsilateral hemisphere at 7 days post-stroke. The extent of oxidized nucleotides was measured by IHC for 8-oxo-dG levels at 24 h post-stroke. (B) IHC and quantification of activated Caspase 3 (aCasp3) and p53 on ipsilateral hemisphere at 7 days post-stroke. n = 3 for HEt, 8-oxo-dG, aCasp3, n = 4 for p53, unpaired Student’s t-test, *p<0.05; ***p<0.001. Scale, 50 μm.

To determine whether reduced oxidative stress may account for the neuroprotective phenotype in *Smad1*-deficient mice, we conducted *in vivo* HEt assays, which showed a 50% decrease in the ROS levels on the ischemic hemisphere in mutant mice, and a 35% reduction in the number of cells undergoing nucleic acid oxidation by 8-oxo-dG immunostaining compared to controls (140 ± 15 vs. 217 ± 7; p < 0.001; Fig 5A). Significantly fewer apoptotic cells (activated Caspase 3^+^) were present in mutant mice than controls (3 ± 1 vs. 34 ± 7 per field examined; p < 0.001; Fig 5B), which is consistent with reduced ROS levels. Similarly, p53 induction after tMCAO was also significantly attenuated in mutants in comparison to controls (21 ± 9 vs. 132 ± 15 p53^+^ cells; p<0.001; Fig 5B). The reduced stroke severity in mutant mice is unlikely a result of major differences in vasculature since immunostaining for CD31, an endothelial marker, showed no detectable differences in the density or distribution of microvasculature (S5D Fig). Examination of the macrovascular network on the cortical surface and circle of Willis at the ventral skull base also revealed no differences between genotypes (S5E Fig). This is in accordance with the absence of Cre-mediated recombination in endothelial cells in our cKO mice (S2B Fig). Furthermore, we monitored cerebral blood flow (CBF) using a laser Doppler Flowmetry probe, and confirmed that mutant and control mice exhibited a similar profile of reduced CBF during the 1 h tMCAO and return of CBF after reperfusion (S6 Fig). Nonetheless, we cannot exclude the possibility that subtle vascular changes such as differences in permeability of blood brain barrier (BBB) or neurovascular niche may contribute to the neuroprotective phenotype observed in the *Smad1* cKO mice, a topic worthy future investigation.

## Discussion

Here, we demonstrate that *Smad1* deletion in the CNS confers neuroprotection in a cerebral I/R injury model in both young and older mice, thus establishing a novel link between Smad1 and stroke pathophysiology. Our model agrees with and further extends previous findings that suppression of BMP/Smad signaling is associated with reduced apoptosis and inflammation following myocardial infarction.

The initial neurological deficits were comparable between wildtype and *Smad1* cKO mice after transient MCAO, indicating similar extent of primary ischemic injury. This is also consistent with a similar degree of reduction in cerebral blood flow during the occlusion of the MCA in both groups (S6 Fig). However, mice with *Smad1* ablation in the CNS showed better neurological recovery at 7 days post-tMCAO than control animals. This occurs at a time point before cortical restructuring and axonal rewiring may have taken place. Likewise, in this early time frame, stroke-triggered neurogenesis is unlikely to have contributed significantly to functional recovery. Thus, the observed neuroprotective phenotypes in *Smad1* cKO mice at 7 day post-stroke are more likely a result of reduced secondary injury than enhanced neural repair. Secondary injury is well known to exacerbate immediate ischemic damage by release of cytokines, ROS/RNS, and apoptotic mediators. Indeed, Smad1-deficient mice exhibited a significant attenuation of reactive astrocytosis and neuroinflammation—two potent sources of secondary injury.

The mechanisms by which Smad1 deletion results in attenuated glial responses and neuronal protection after stroke remain to be determined. We observed an enhanced anti-oxidant capacity in *Smad1* deficient astrocytes, which may account for the protective phenotypes after tMCAO. However, since *Smad1* is also deleted in neurons in the mutant mice, it is possible that it may lead to direct neuronal protective effects against ischemic stress. The relative contribution of protective mechanisms in neurons versus glial cells needs to be further resolved in future studies using cell-type specific Cre recombinase lines. Furthermore, although Nestin-Cre mediated recombination does not appear to have occurred to a large extent in endothelial cells and no major vascular abnormalities were observed in *Smad1* cKO mice, subtle changes in the neurovascular niche and permeability of BBB to immune cells remain a possibility, and may contribute to the protective phenotype observed in the *Smad1* cKO mice after tMCAO. Additional in-depth investigation into the structure and integrity of the cerebral vasculature and the usage of cell-type-specific Cre lines are imperative to address the potential contribution of vascular differences to stroke protection. *Smad1* deletion may also engage other immediate or long-term protective mechanisms, such as reducing cellular metabolism or promoting synaptic plasticity [29], which need to be further explored. It is also noteworthy that the current study combines data from both sexes, thus, does not address gender effects in the *Smad1* cKO mice after stroke, a topic worthy of future investigation.

Our data show that *Smad1* deletion affects several aspects of astrocytic function, which may contribute to neuroprotection against stroke: i) GFAP expression is elevated in astrocytes in uninjured *Smad1* cKO mice. GFAP is a reliable marker for reactive astrocytes [22], thus the baseline upregulation of GFAP in astrocytes in conjunction with the associated hypertrophic morphological changes implies increased baseline levels of astrocyte reactivity, perhaps in response to cumulative physiological stressors. This may lead to enhanced antioxidant capacity in astrocytes as a result of the priming effect against subsequent pathological stressors, akin to the well-described ischemic preconditioning [25]. ii) Absence of Smad1 activity may directly lead to enhanced antioxidant capacity in astrocytes, for the following reason: astrocytes used for oxidative stress test were derived from neonatal pups (Fig 1F), thus they are unlikely to have had sufficient time to experience a potential priming effect (however, extrapolation of the findings from cultured neonatal primary astrocytes to adult astrocytes in vivo requires caution). Notably, *Smad1* deletion does not seem to prevent astrocytes from mounting a reaction to CNS injury, as in areas of severe ischemia, such as in the striatal stroke core, robust reactive astrocytosis did occur in both mutant and control mice (Fig 2A). It is also worth mentioning that although BMP promotes astrocyte differentiation during development [30], *Smad1* deletion does not appear to affect general astrocyte development. This may be related to compensatory roles of other Smads or that BMP may signal through non-canonical, Smad1-independent pathways to promote astrocyte differentiation.

Our current study extends previous work on transcription factors that regulate a broad range of ischemia-induced genes in a cell-type specific and time-dependent manner, including nuclear factor-κB (NF-κB), HIF-1α, Nrf2, and CREB [31–34]. Downstream targets of Smad1 in the setting of cerebral ischemia remain to be identified, but may include regulators of redox homeostasis, as ROS levels and nucleotide oxidation are lower in ischemic tissue in the absence of Smad1. This is in agreement with the reported involvement of BMP/Smad1 signaling in redox homeostasis through NOX activation in O-2A progenitor cells [35], cardiomyocytes [9], vascular epithelial cells [36] and vascular smooth muscle cells [37]. Notably, in the current study, we did not observe significant differences in the transcription of nine pro- and antioxidant genes examined in uninjured cortical tissue or at 3 days post-stroke between the groups (S4B Fig). Future cell-type specific and time-dependent studies on the protein level or on other redox-associated genes may provide further insight. In addition, *Smad1* deletion may affect other target genes such as p53. Although we observed that p53 induction after stroke was attenuated in Smad1-deficient mice, the mechanism by which Smad1 is linked to p53 induction after cerebral I/R injury remains to be clarified.

Finally, in cerebral ischemia, Smad1 may mainly function as a downstream effector of canonical BMP signaling, or alternatively, as a mediator of other stroke-induced signaling pathways, such as brain-derived neurotrophic factor (BDNF) or GSK3β signaling [38, 39]. Consistent with our results and in support of a role of Smad1 in mediating canonical BMP signaling in stroke, Noggin, an endogenous BMP antagonist, has been shown to confer protection against ischemic brain injury. Overexpression of Noggin in a transgenic ischemic model leads to smaller infarct volumes and attenuated motor deficits after cortical focal stroke [40]. Administration of Noggin-expressing bone marrow stromal cells (BMSCs) also enhances brain repair after MCAO [41]. On the other hand, intraventricular injection of BMP7 reportedly facilitates motor recovery after stroke [42], although the effect is probably through enhanced proliferation of neural progenitors [43], which takes place in a much later timeframe than that of the current study. Whether Smad1 is also involved in activating neural progenitors after cerebral ischemia awaits further study. Since the extent of neurogenesis from the subventricular zone (SVZ) is a function of stroke size, a simple comparison of ischemia-triggered neurogenesis in our mutant mice may be confounded by the more limited tissue injury in *Smad1* cKO mutants, hence, selective ablation of Smad1 in SVZ cells will be required.

Our finding of a potential link between astrocyte phenotype (GFAP upregulation and hypertrophic morphology) and stroke protection raises a tantalizing possibility that Smad1 ablation in the CNS may mimic the well-known preconditioning effect in raising the baseline levels of astrocyte reactivity. This working model requires further mechanistic studies to identify the trigger that leads to enhanced astrocyte reactivity following *Smad1* deletion in the CNS. Nonetheless, our study opens door for future exploration to determine whether inhibition of BMP/Smad1 signaling by small-molecule inhibitors, such as dorsomorphin [44], shortly after stroke would confer neuroprotection, or whether for high-risk patients administering a selective BMP/Smad inhibitor preemptively would serve as a preventative measure against stroke. Our study also raises the possibility that *Smad1* deletion may confer protection against other CNS stressors that are associated with aging and neurodegenerative disorders.

## Conclusions

We have demonstrated a neuroprotective phenotype in Smad1-deficient mice following cerebral I/R injury. Developing strategies to inhibit the Smad1-mediated pathway may represent a novel direction for stroke protection.

## Supporting Information

S1 Fig Nestin-Cre recombination in Rosa26-YFP reporter line. Fluorescent images of YFP (highlighted in green with anti-GFP antibody) and cell-type specific markers (red) in Nestin-Cre; Rosa26-YFP reporter mice showing Nestin-Cre recombination in neurons (NeuN), astrocytes (GFAP, S100B), and oligodendrocytes (Olig2, photo taken in the corpus callosum). DAPI counterstaining is in blue. Scale, 50 μm.
(TIF)

S2 Fig No detectable Nestin-Cre recombination in microglia and endothelial cells. (A) Fluorescent images of YFP (highlighted in green with anti-GFP antibody) and cell-type specific markers for microglia (Iba1 in red) in Nestin-Cre; Rosa26-YFP reporter mice showing no detectable Nestin-Cre recombination in microglia in cortex. (B) Rosa26-YFP reporter line showed that Nestin-Cre recombination was not detectable in CD31^+^ endothelial cells (arrows) in cortex (top panels) or corpus callosum area (bottom panels). Scale, 50 μm.
(TIF)

S3 Fig Elevated reactive state of astrocytes in *Smad1* cKO mice. (A) Body weights of control and *Smad1* cKO mice were not significantly different at any age. (B) Immunofluorescence images of cortex in uninjured mice showed upregulated GFAP in the *Smad1* cKO mice compared to controls. Enlarged images of boxed areas in (A) are shown on the right, demonstrating that GFAP^+^ astrocytes formed extensive contacts with cerebral microvasculature in both control and *Smad1* cKO mice. Scale, 100 μm (B) and 50 μm (enlarged images on the right).
(TIF)

S4 Fig Inflammatory and redox gene expression profiles in post-stroke cortex. (A-B) qRT-PCR results did not reveal statistically significant differences between *Smad1* cKO and control littermates in the mRNA levels of different cytokines (A) or redox genes (B) in cortical tissues before or after stroke (3 days post-MCAO). The pro- or antioxidant genes include: pro-oxidant enzymes NADPH oxidases (NOX)-1, -2, and -4 [45], ROS-scavengers superoxide dismutase (SOD)-1 and -2 [46], sulfiredoxin (*Srxn1*) [47], glutamate–cysteine ligase catalytic subunit (Gclc, the first rate-limiting enzyme of glutathione synthesis) [48], Nrf2 [49] and heme oxygenase 1 (*HO-1)*.
(TIF)

S5 Fig Behavioral assessment and cerebral vasculature in *Smad1* cKO mice. (A) At 7 days post-stroke, control mice exhibited ptosis of the left eyelid (yellow arrow) and weakness in the right forelimb, which was often in paretic spastic posture (green arrowhead). Both symptoms were less severe in *Smad1* cKO mice. (B) In 8–10 week old cohorts, neurological deficit scores showed that both *Smad1* cKO and control groups recovered to a similar extent by 1 and 3 months post-stroke (n = 1 female and 3 males in the control cohort and 1 female and 2 males in the *Smad1* cKO cohort. (C) Older *Smad1* cKO mice (8–10 months old) also showed significant improvement in neurological function at 7 days but not 1 day post-stroke (p = 0.03). (D) CD31 staining did not reveal differences in microvasculature in *Smad1* cKO mice. (E) Surface vasculature (top) and Circle of Willis (bottom) appeared similar in both groups, as revealed by Bromophenol blue dye infusion.
(TIF)

S6 Fig Cerebral blood flow in the transient MCAO model. Laser Doppler flowmetry probe was used for cerebral blood flow (CBF) tracing. After ligation of the left common carotid artery (LCC), there was a 50% drop of CBF. MCA occlusion after insertion and advancement of the suture led to a further decrease by over 80–90% of baseline CBF. After 1 h MCAO, the suture was removed and the left common carotid artery was permanently ligated to prevent bleeding, resulting in a slow return of CBF to approximately 50% of baseline levels. The CBF tracing showed a similar response of CBF in control and *Smad1* cKO mice during MCAO and subsequent reperfusion.
(TIF)
