# Supplementary figures and images for: Attenuation of Cerebral Ischemic Injury in Smad1 Deficient Mice
Source: PLoS One. 2015 Aug 28;10(8):e0136967. doi: 10.1371/journal.pone.0136967 (PMC4552810; doi:10.1371/journal.pone.0136967)

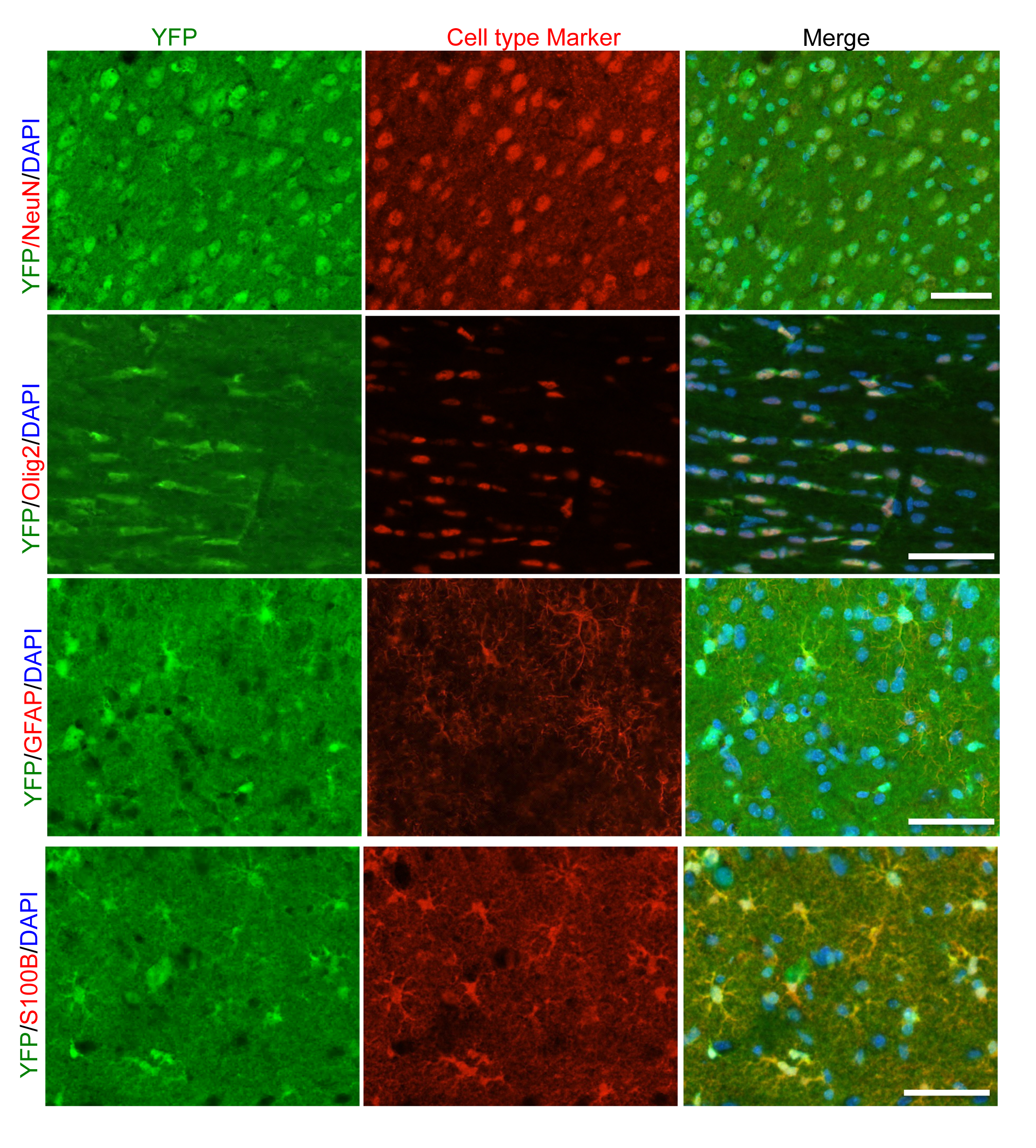

Supplement: S1 Fig — Fluorescent images of YFP (highlighted in green with anti-GFP antibody) and cell-type specific markers (red) in Nestin-Cre; Rosa26-YFP reporter mice showing Nestin-Cre recombination in neurons (NeuN), astrocytes (GFAP, S100B), and oligodendrocytes (Olig2, photo taken in the corpus callosum). DAPI counterstaining is in blue. Scale, 50 μm. (TIF) [file pone.0136967.s001.tif]

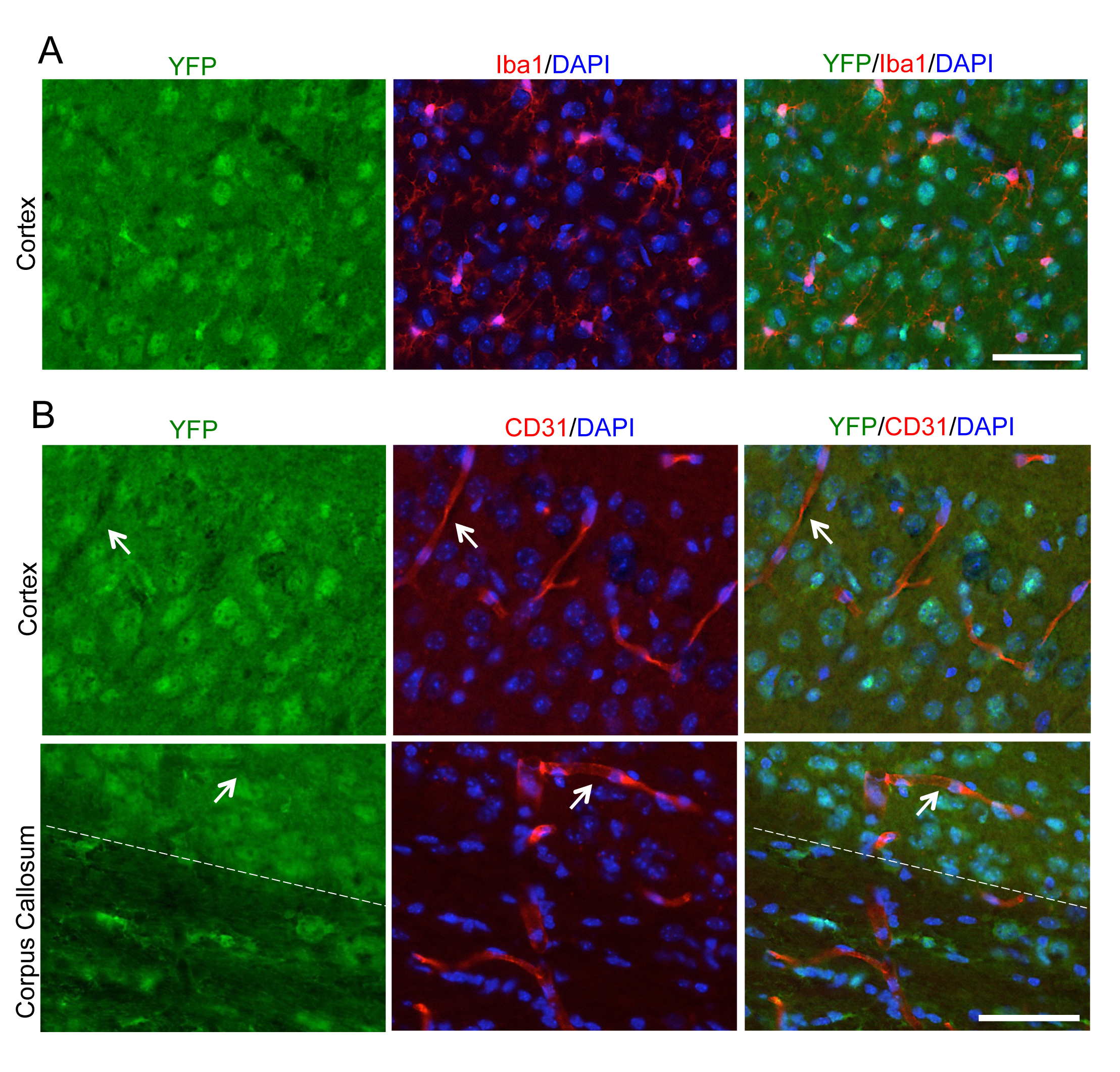

Supplement: S2 Fig — (A) Fluorescent images of YFP (highlighted in green with anti-GFP antibody) and cell-type specific markers for microglia (Iba1 in red) in Nestin-Cre; Rosa26-YFP reporter mice showing no detectable Nestin-Cre recombination in microglia in cortex. (B) Rosa26-YFP reporter line showed that Nestin-Cre recombination was not detectable in CD31+ endothelial cells (arrows) in cortex (top panels) or corpus callosum area (bottom panels). Scale, 50 μm. (TIF) [file pone.0136967.s002.tif]

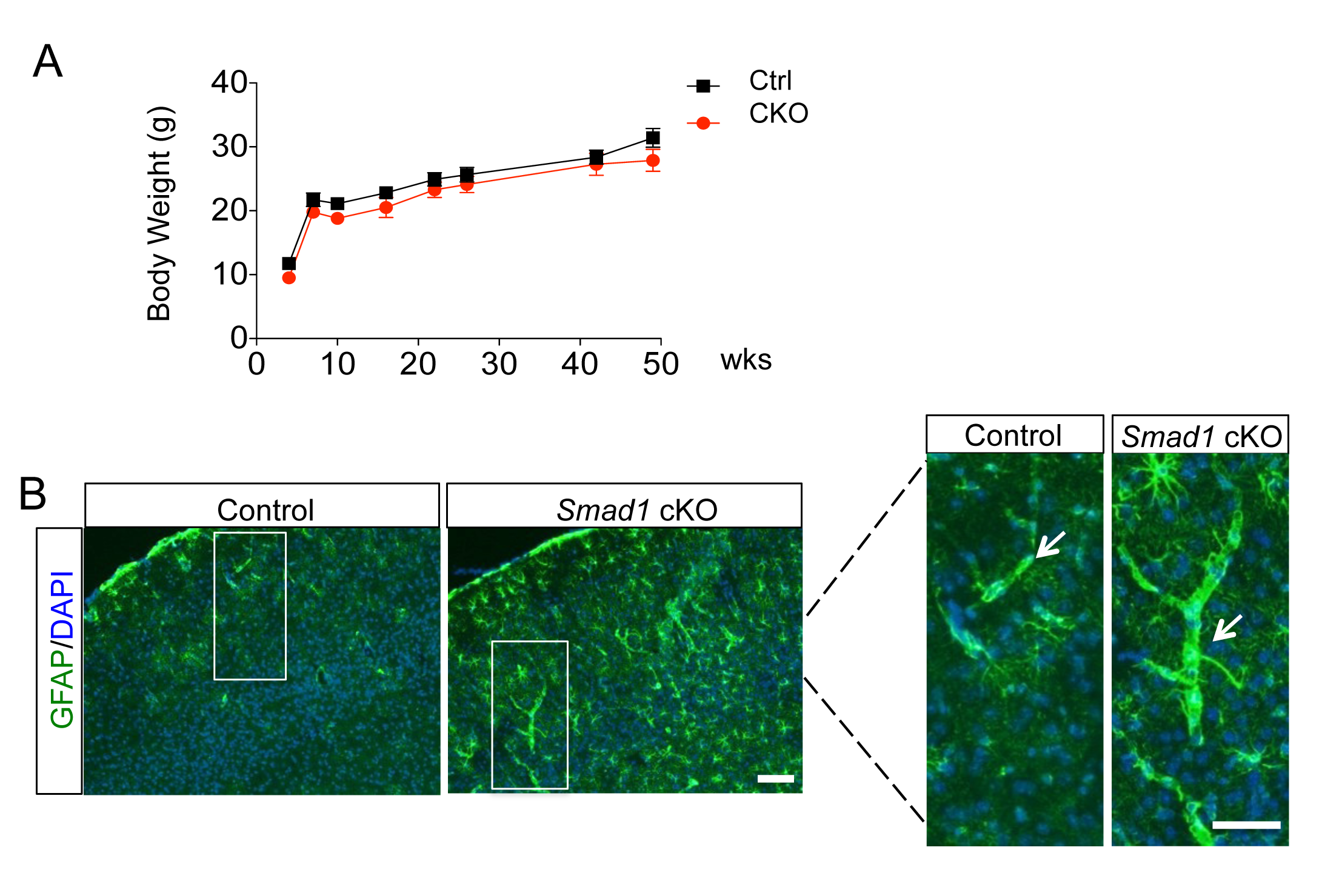

Supplement: S3 Fig — (A) Body weights of control and Smad1 cKO mice were not significantly different at any age. (B) Immunofluorescence images of cortex in uninjured mice showed upregulated GFAP in the Smad1 cKO mice compared to controls. Enlarged images of boxed areas in (A) are shown on the right, demonstrating that GFAP+ astrocytes formed extensive contacts with cerebral microvasculature in both control and Smad1 cKO mice. Scale, 100 μm (B) and 50 μm (enlarged images on the right). (TIF) [file pone.0136967.s003.tif]

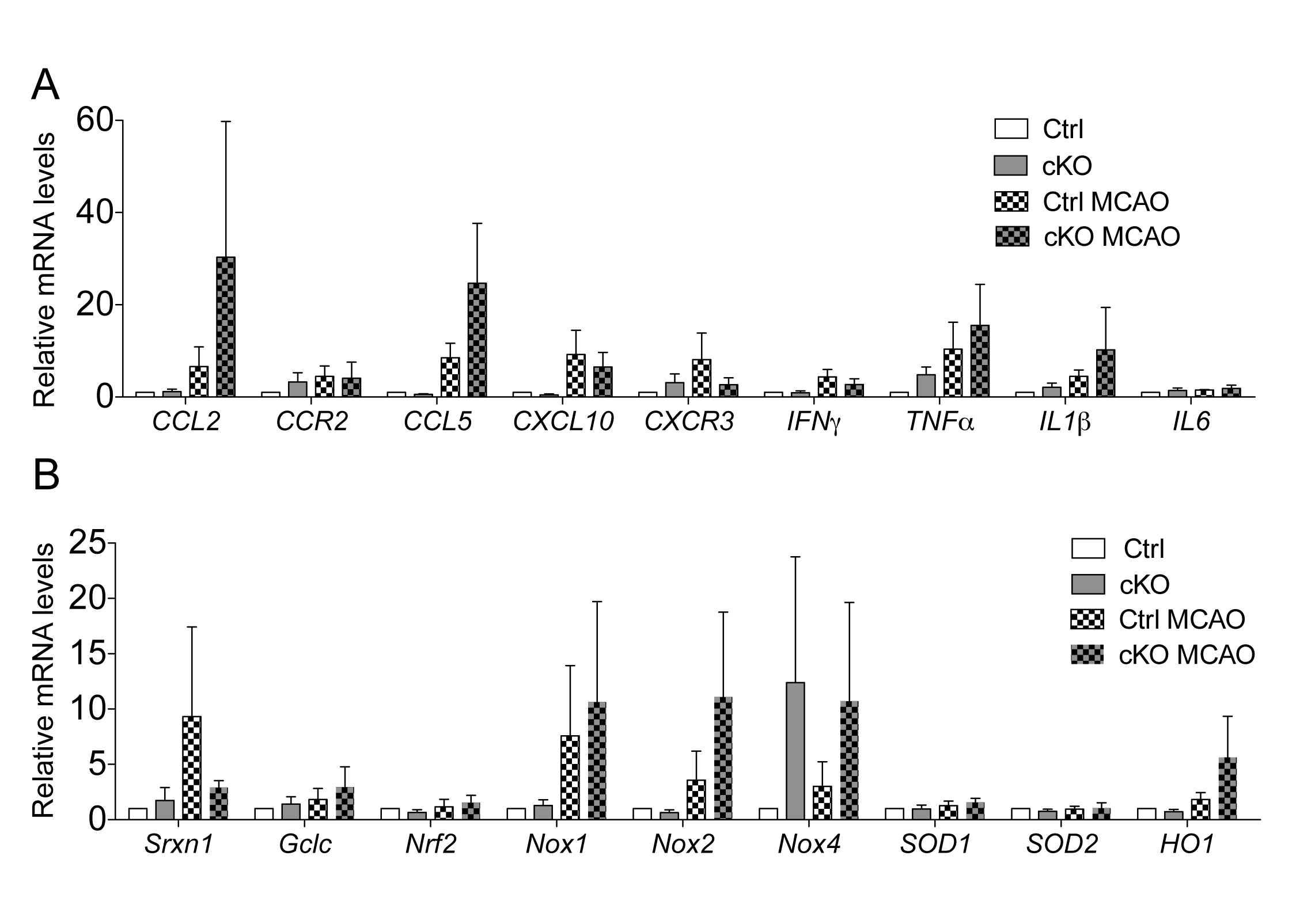

Supplement: S4 Fig — (A-B) qRT-PCR results did not reveal statistically significant differences between Smad1 cKO and control littermates in the mRNA levels of different cytokines (A) or redox genes (B) in cortical tissues before or after stroke (3 days post-MCAO). The pro- or antioxidant genes include: pro-oxidant enzymes NADPH oxidases (NOX)-1, -2, and -4 [45], ROS-scavengers superoxide dismutase (SOD)-1 and -2 [46], sulfiredoxin (Srxn1) [47], glutamate–cysteine ligase catalytic subunit (Gclc, the first rate-limiting enzyme of glutathione synthesis) [48], Nrf2 [49] and heme oxygenase 1 (HO-1). (TIF) [file pone.0136967.s004.tif]

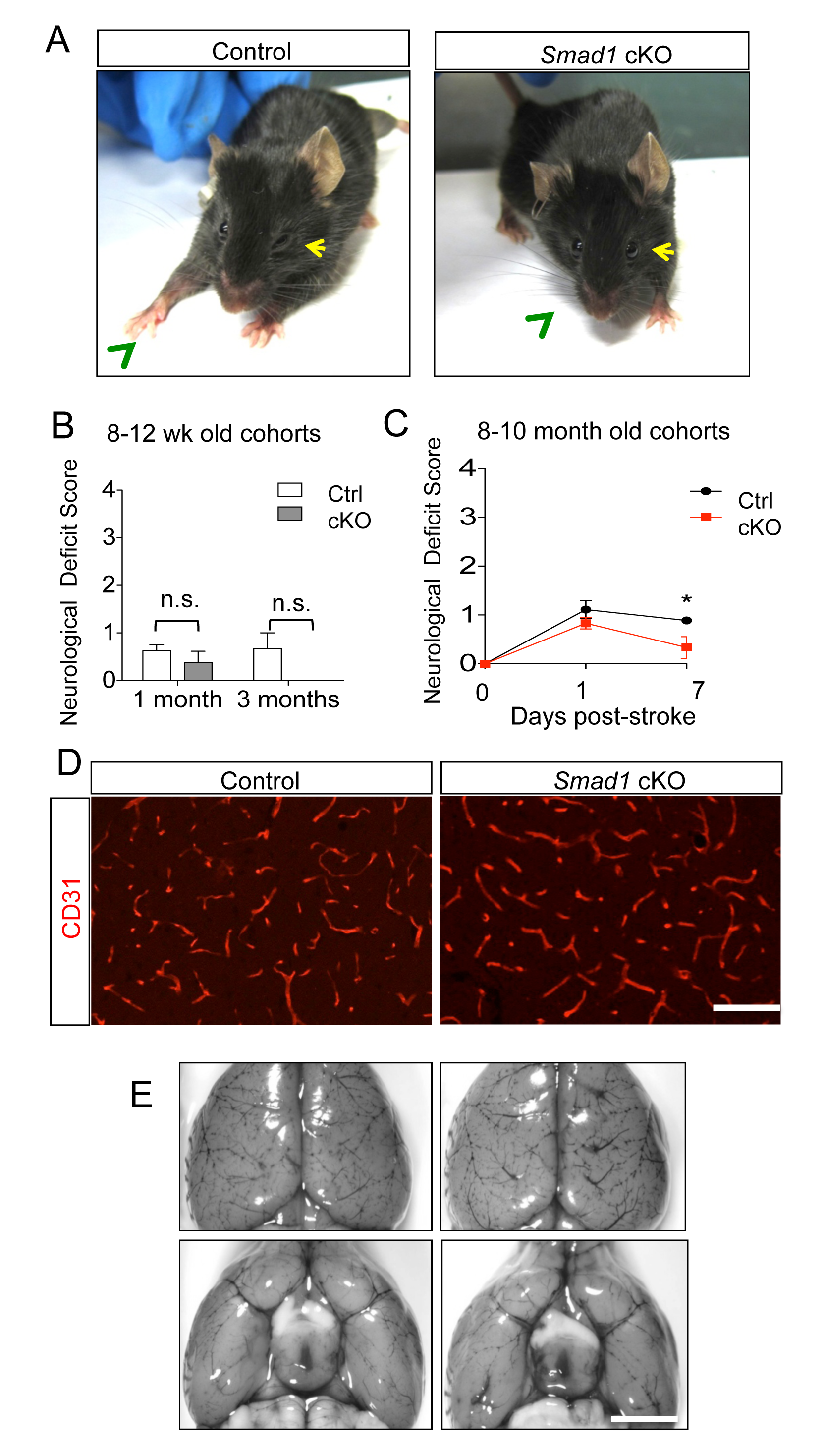

Supplement: S5 Fig — (A) At 7 days post-stroke, control mice exhibited ptosis of the left eyelid (yellow arrow) and weakness in the right forelimb, which was often in paretic spastic posture (green arrowhead). Both symptoms were less severe in Smad1 cKO mice. (B) In 8–10 week old cohorts, neurological deficit scores showed that both Smad1 cKO and control groups recovered to a similar extent by 1 and 3 months post-stroke (n = 1 female and 3 males in the control cohort and 1 female and 2 males in the Smad1 cKO cohort. (C) Older Smad1 cKO mice (8–10 months old) also showed significant improvement in neurological function at 7 days but not 1 day post-stroke (p = 0.03). (D) CD31 staining did not reveal differences in microvasculature in Smad1 cKO mice. (E) Surface vasculature (top) and Circle of Willis (bottom) appeared similar in both groups, as revealed by Bromophenol blue dye infusion. (TIF) [file pone.0136967.s005.tif]

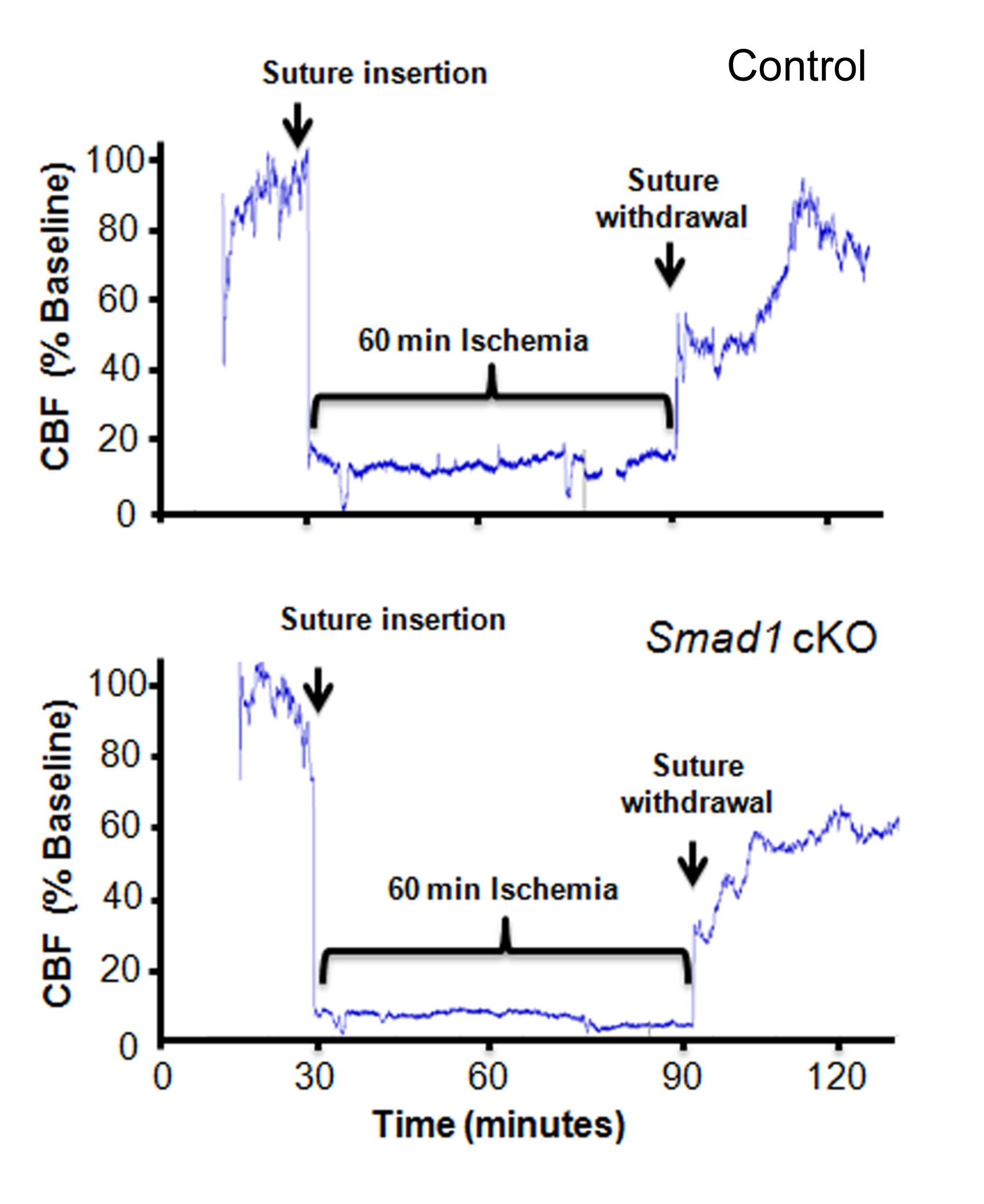

Supplement: S6 Fig — Laser Doppler flowmetry probe was used for cerebral blood flow (CBF) tracing. After ligation of the left common carotid artery (LCC), there was a 50% drop of CBF. MCA occlusion after insertion and advancement of the suture led to a further decrease by over 80–90% of baseline CBF. After 1 h MCAO, the suture was removed and the left common carotid artery was permanently ligated to prevent bleeding, resulting in a slow return of CBF to approximately 50% of baseline levels. The CBF tracing showed a similar response of CBF in control and Smad1 cKO mice during MCAO and subsequent reperfusion. (TIF) [file pone.0136967.s006.tif]
